# SFRP2 Potentiates Metastasis of Colon Cancer by Enhancing Snai1 Protein Stability via USP11

**DOI:** 10.1111/jcmm.71318

**Published:** 2026-09-11

**Authors:** Yanshen Kuang, Xin Liu, Mu Ke, Zhijie Chang, Baoqing Jia, Bing Li, Honggang Qian

**Affiliations:** ^1^ Department of General Surgery The First Medical Centre, Chinese PLA General Hospital Beijing China; ^2^ State Key Laboratory of Membrane Biology, School of Medicine, National Engineering Laboratory for Anti‐Tumor Therapeutics Tsinghua University Beijing China; ^3^ Key Laboratory of Carcinogenesis and Translational Research, Department of Hepato‐Pancreato‐Biliary Surgery Peking University Cancer Hospital Beijing China

**Keywords:** colon cancer, metastasis, PI3K/AKT/mTOR, SFRP2, Snai1, USP11

## Abstract

Metastatic colon cancer remains a significant clinical challenge, often associated with poor patient prognosis. Recent studies have identified the secreted frizzled‐related protein 2 (SFRP2) as a key player in this malignancy, showing its overexpression in metastatic cases. This research aimed to elucidate the molecular mechanisms by which SFRP2 influences colon cancer progression. We employed in vitro assays to assess the effects of SFRP2 on cell migration and invasion, revealing that SFRP2 significantly enhances these processes without affecting cell proliferation. Mechanistically, we found that SFRP2 interacts with Snai1, a transcription factor known to promote epithelial‐mesenchymal transition (EMT), thereby stabilizing Snai1 protein levels. Furthermore, SFRP2 was shown to inhibit Snai1 ubiquitination through the deubiquitinase USP11, leading to increased Snai1 activity. Notably, Snai1 also positively regulates SFRP2 transcription, establishing a feedback loop that amplifies their expression. Our findings indicate that the SFRP2‐Snai1 axis promotes colon cancer cell migration and invasion via the phosphoinositide 3‐kinase (PI3K)/protein kinase B (AKT)/mammalian target of rapamycin (mTOR) signalling pathway. In vivo experiments further confirmed that the SFRP2‐Snai1 interaction significantly enhances metastatic potential. Collectively, these results suggest that targeting the SFRP2‐Snai1 signalling pathway may provide a novel therapeutic strategy for metastatic colon cancer.

## 1 Introduction

Colon cancer is one of the leading causes of cancer‐related morbidity and mortality worldwide, with an estimated incidence of over 1.9 million new cases and approximately 935,000 deaths annually [1, 2, 3]. The metastatic potential of colon cancer significantly complicates treatment, as metastasis is responsible for the majority of cancer‐related deaths [4, 5, 6]. Current clinical therapies, including surgery, chemotherapy and targeted therapies, often face challenges due to the heterogeneity of the disease and the development of drug resistance [7, 8]. Moreover, the lack of effective biomarkers for early detection and monitoring of metastasis further exacerbates the situation, leading to poor prognoses for many patients. Further investigation of the molecular mechanisms underlying colon cancer progression and metastasis is crucial for developing more effective therapeutic strategies.

Secreted frizzled‐related protein 2 (SFRP2) is a member of the SFRP family, which plays a crucial role in modulating Wnt signalling pathways [9, 10]. SFRP2 is known to be involved in various biological processes, including cell proliferation, differentiation and migration. Its function as an antagonist of Wnt signalling has been well‐documented [11, 12, 13, 14, 15]. However, recent studies have revealed that SFRP2 can also act as a crucial role in tumour progression. It was reported that SFRP2 could induce a mesenchymal subtype transition by suppression of SOX2 in glioblastoma [16]. It was shown that downregulation of SFRP2 could facilitate cancer stemness and radioresistance of glioma cells via activating Wnt/β‐catenin signalling [17]. A study reported that SFRP2 could contribute to the invasive and metastatic potential for osteosarcoma [18]. SFRP2 was also found to act as an oncogenic role in the development of p53 mutation‐associated osteosarcoma [19]. SFRP2 was found to be involved in METTL3‐mediated the malignancy of non‐small cell lung cancer [20]. SFRP2 has emerged as a pivotal player in various pathological processes, including cancer progression, fibrosis and metabolic disorders, including contributing to skin fibrosis, regulating epithelial‐mesenchymal transition in colorectal cancer and influencing collagen deposition in dermal development. Elevated levels of SFRP2 are associated with poor clinical outcomes, highlighting its potential as a therapeutic target and prognostic biomarker in conditions such as colorectal cancer and chronic diseases like systemic sclerosis [21]. Despite these insights, the precise mechanisms underlying SFRP2's role in colon cancer metastasis remain inadequately understood, and further research is needed to elucidate its full impact on colon cancer progression and metastasis.

This study aims to elucidate the role of SFRP2 in colon cancer metastasis. Specifically, we investigate the mechanisms by which SFRP2 influences cellular behaviours such as migration and invasion, while also exploring its potential regulatory mechanism interacting with Snai1, a key regulator of epithelial‐mesenchymal transition (EMT). The significance of this research lies in its potential to uncover novel therapeutic targets for metastatic colon cancer. By employing in vitro and in vivo investigations, we seek to provide a comprehensive understanding of the SFRP2‐Snai1 axis and its impact on the phosphoinositide 3‐kinase (PI3K)/AKT/mTOR signalling pathway. Ultimately, this work aims to contribute to the growing body of knowledge regarding the molecular underpinnings of colon cancer progression and to identify actionable insights that could inform future treatment strategies.

## 2 Methods and Materials

### 2.1 Bioinformatics Analysis

Publicly available microarray datasets were downloaded from GEO (https://www.ncbi.nlm.nih.gov/geo/) with chip annotation data. All analyses were carried out using R (4.2.1) package. GEO: GSE40367 [22, 23]. Difference analysis was performed by GEOquery[2.64.2], limma[3.52.2], ggplot2[3.3.6], ComplexHeatmap[2.13.1] [24, 25]. Gene Set Enrichment Analysis was performed using clusterProfiler[4.4.4] [26, 27]. The following public databases were searched: the Cancer Genome Atlas (TCGA) database (https://gdac.broadinstitute.org/) and Kaplan–Meier Plotter (http://kmplot.com/analysis/). BioGRID (http://thebiogrid.org/) was used for protein–protein interaction analysis.

### 2.2 Cell Culture and Transfection

HCT116 and LoVo were purchased from Wuhan Pricella Biotechnology Co. Ltd. LoVo cells were cultured in Ham's F‐12 K medium supplemented with foetal bovine serum (FBS) and 100 U/mL penicillin G and 100 μg/mL streptomycin sulphate (P.S.). HCT116 was cultured in McCoy's 5A with 10% FBS and 1% P.S. The SFRP2 or Snai1 overexpressing plasmids or shRNAs were transfected with lipo2000. The targeted sequences were listed in Table S1.

### 2.3 Western Blotting

Total protein was isolated using the RIPA lysis buffer (Beyotime Institute of Biotechnology, Haimen, China), and the protein concentration was quantified using a bicinchoninic acid protein assay kit (Beyotime Biotechnology, Shanghai, China). Proteins (30 μg) were resolved using 10% SDS‐PAGE, transferred onto a nitrocellulose membrane and blocked with non‐fat milk. The membranes were incubated with primary antibodies against E‐cadherin (1:1000, ABclonal, A3044), N‐cadherin (1:1000, ABclonal, A0433), Vimentin (1:1000, ABclonal, A19607), SFRP2 (1:1000, ABclonal, A5383), PI3K (1:1000, CST, #13,666), p‐PI3K (1:1000, CST, #17,366), Akt (1:1000, CST, #4685), p‐Akt (1:1000, CST, #4060), mTOR (1:1000, CST, #2983), p‐mTOR (1:1000, CST, #2971), GAPDH (1:2000, ABclonal, A19056) overnight at 4°C, followed by the α‐mouse or α‐rabbit‐HRP secondary antibodies. Proteins were visualized using an enhanced chemiluminescence (ECL) kit (Beyotime). Bands were quantified by the ImageJ software.

### 2.4 Quantitative Real‐Time PCR (RT‐qPCR)

RNA was extracted using the TRIzol reagent, then was reverse transcribed to form cDNA. Real‐time quantitative PCR reactions were then performed on an ABI Prism 7500 System (Applied Biosystems, Carlsbad, CA, USA) with TransStart Top Green qPCR SuperMix (Transgen, Beijing, China). The relative expression of the target genes normalized to GAPDH was calculated using the 2^−ΔΔCt^ method. The sequences of primers are listed in Table S1.

### 2.5 Immunohistochemistry (IHC)

The samples derived from patients with colon cancer were obtained from the First Medical Centre, Chinese PLA General Hospital (S2022‐610‐01). Informed consent was secured from all participants, and authorization for the utilization of tumour specimens was provided by the medical ethics committee of the First Medical Centre of Chinese PLA General Hospital. Tumours were preserved in 4% paraformaldehyde overnight at 4°C. Subsequently, tumours were encased in paraffin and sectioned at 5 μm. Immunohistochemistry was conducted in accordance with standard protocols. Following incubation with primary antibodies, HRP‐conjugated Goat anti‐rabbit IgG were employed, Representative images from tumours of each group were utilized to quantify the IHC signals of SFRP2, The IHC sections were further scanned and analysed with the ImageJ.

### 2.6 Seperation of Low and High Metastatic LoVo Cells

The establishment of cancer cell sublines with different metastatic abilities was reported previously [28, 29]. The in vitro invasion model was constructed using chambers coated with Matrigel (BD biosciences, 356,234), which were inserted into 6‐well plates. LoVo cells were cultured with Ham's F‐12 K not containing FBS for starvation for 24 h before digestion. A total of 5 × 10^5^ cells suspended in Ham's F‐12 K without FBS was added into the upper chamber. The down chamber was added with Ham's F‐12 K with 20% (v/v) FBS. 24 h incubation, cells from upside membrane (UM) and downside membrane (DM) of the chamber were harvested and cultured, respectively. After three rounds, only the first collected upside membrane cells were identified as the low metastatic cells (UM). The cells penetrating through the membrane were called the high metastatic cells (DM). The collected cells were used for western blot analysis.

### 2.7 Cell Counting Kit‐8 (CCK‐8)

Cell proliferation assay was performed using CCK‐8 (Beyotime). HCT116 or LoVo cells were transfected as indicated then seeded into a 96‐well plate at a density of 2 × 10^3^ for 24, 48 and 72 h. A CCK‐8 solution (10 μL) was added to each well and incubated for about 2 h. The absorbance at 450 nm was measured using a microplate reader.

### 2.8 Transwell

Cell invasion assays were conducted in 24‐well plates with the Matrigel (BD, 356234)‐coated inserts (8.0 mm pore). HCT116 or LoVo cells (1 × 10^5^) were deposited into the upper chamber, while 600 μL of complete culture medium with 10% FBS was added to the lower chamber. Cells were incubated for 24 h and the upper compartment of cells were removed. The invading cells were counted in five random fields at ×400 magnification. For the migration assays, a total of 5 × 10^4^ colon cancer cells were placed into 8.0 mm pore inserts devoid of Matrigel. The migration duration was 12 h. The migration method was performed as mentioned above.

### 2.9 Co‐Immunoprecipitation (Co‐IP) Assay

The transfected cells were collected in RIPA lysis buffer containing a phosphorylase inhibitor, incubated for 30 min on ice and then centrifuged at 12,000 × g for 10 min. The protein A/G agarose beads were treated with antibodies targeting SFRP2, Snai1, Flag, HA or a negative control IgG, overnight at 4°C with rotation. The complexes were rinsed with elution buffer and heated for 10 min, followed by Western blot analysis.

### 2.10 Luciferase Assay

The SFRP2 promoter and the mutant SFRP2 promoter were inserted into the luciferase reporter gene vector pGL4, respectively. Cells were cultivated in 24‐well culture plates and cotransfected with combinations of the respective reporter plasmid and the specified plasmids in each experiment following the standard protocol (https://www.promega.com.cn/resources/protocols/). Luciferase activity was measured using a Dual‐Luciferase Report Assay (Promega) system in accordance with the manufacturer's guidelines.

### 2.11 Chromatin Immunoprecipitation (ChIP)

ChIP assays were performed using the ChIP Kit (Univ‐bio) in accordance with the manufacturer's instructions. Cells were cultured to 80%–90% confluence and crosslinked with 1% formaldehyde for 10 min before adding 0.125 M glycine for 5 min. The crosslinked DNA was cleaved to fragmented DNA ranging from 200 to 1000 bp. Antibody against Snai1 or IgG was performed to immunoprecipitate DNA‐associated complexes. qRT–PCR was used to identify the Snai1‐binding site within the SFRP2 promoter regions.

### 2.12 In Vivo Metastasis Assays

All animal experimental procedures were approved by the Institutional Animal Care Committees of the First Medical Centre of the Chinese PLA General Hospital (23‐CZJ4.G24‐1). For the peritoneal metastatic xenograft model, cells were transduced with firefly luciferase reporter lentivirus and then with vector or SFRP2 or shNC or shSFRP2 lentiviruses. 2 × 10^6^ stable cells resuspended in sterile PBS were injected into the abdominal cavity of NOD/Shi‐scid/IL‐2Rgamma null (NSG) 8‐weeks‐old male mice, respectively. After approximately 4 weeks, the bioluminescence imaging was used to evaluate the liver metastasis distribution. The substrate luciferin (30 mg/mL, at a dosage of 150 mg/kg body weight) was administered into the intraperitoneal cavity about 15 min prior to imaging. Mice were anaesthetised with isoflurane/oxygen and positioned on the imaging platform. The images of metastasis‐bearing livers were photographed by a fluorescent dissection microscope equipped with a colour camera.

### 2.13 Statistical Analysis

All data are presented as the mean ± SD and analysed by Student's *t*‐test or one‐way ANOVA. Statistical analysis was performed using Graphpad Prism (Version 9.4.0). Correlations were evaluated by using a Pearson correlation test. *p* values < 0.05 were considered statistically significant.

## 3 Results

### 3.1 SFRP2 Is Overexpressed in Metastatic Colon Cancer and Associated With a Poor Prognosis

**FIGURE 1:**
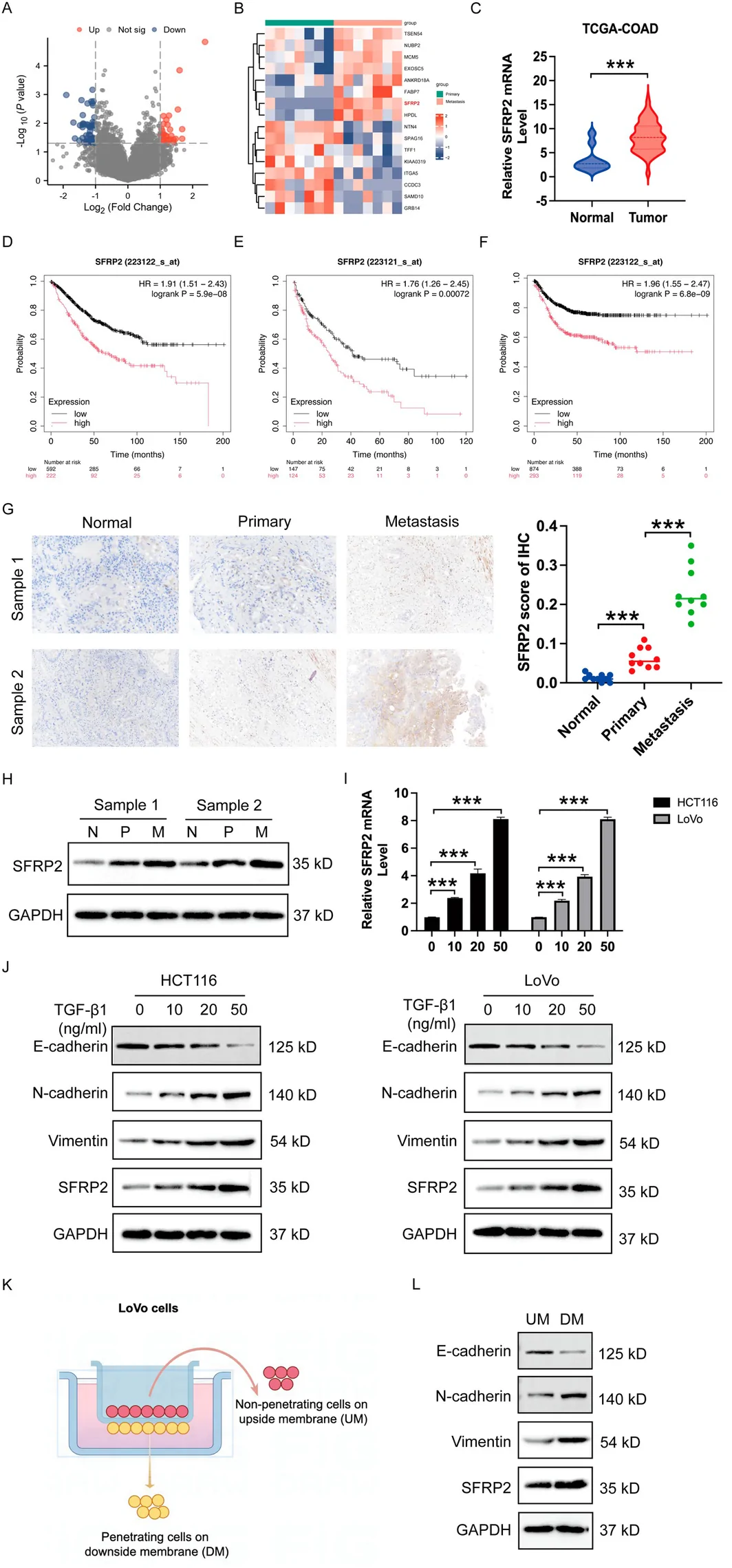
SFRP2 is increased in metastatic colon adenocarcinoma and TGF‐beta induced colon cancer cells. (A) Volcano plot was with differentially expressed genes between metastatic colon cancer and primary cancer in GSE40367. (B) Heatmap showing expression levels of eight up‐regulated and eight down‐regulated different expressed genes in metastatic colon cancer compared to primary colon cancer. (C) Expression of SFRP2 was increased in colon cancer compared to normal tissues in TCGA. (D‐F) Kaplan–Meier plotter of SFRP2 in overall survival (OS), recurrence‐free survival (RFS) and post‐progression survival (PPS) for colon cancer. (G) Representative images of immunohistochemical analysis for SFRP2 in colon cancer samples. IHC score of SFRP2 in normal, primary and metastasis colon cancer tissues. (H) Western blot analysis of the expression of SFRP2 in normal, primary and metastasis colon cancer tissues. (I and J) Western blot analysis of expression of SFRP2, E‐cadherin, N‐cadherin and Vimentin in HCT116 and LoVo cells with different concentrations of TGF‐β1 (0, 10, 20, 50 ng/mL) treatment. ***p < 0.001.

To verify metastasis associated genes of colon cancer, we analysed differences between primary and metastatic colon cancer tissues based on the expression profile data of GEO GSE40367. Volcano plot showed that SFRP2 was significantly increased among differentially expressed genes in metastatic colon cancer tissues compared to primary tumour tissues (Figure 1A). Heatmap also showed that SFRP2 was upregulated in metastatic colon cancer tissues (Figure 1B). Consistently, SFRP2 expression was also elevated in colon cancer tissues compared to normal tissues in TCGA colon cancer (Figure 1C). Additionally, Kaplan–Meier Plotter online analysis showed that higher SFRP2 level was associated with poor overall survival (OS), recurrence free survival (RFS) and post‐progression survival (PPS) (Figure 1D–F). Furthermore, immunohistochemistry result showed that SFRP2 was highly expressed in the metastatic and primary tumour tissues compared to normal tissues and the highest expressed in metastatic tumour tissues (Figure 1G), consistent with western blot analysis (Figure 1H). Furthermore, we also evaluated the expression of SFRP2 in TGF‐β‐mediated metastasis cell model. We found that SFRP2 expression was elevated with increased concentration of TGF‐β treatment, along with the decreased expression of E‐cadherin and increased expression of N‐cadherin and Vimentin (Figure 1I,J). Moreover, we performed the isolation of high metastatic LoVo cells from down membrane (DM) and low metastatic LoVo cells from upper membrane (UM) (Figure 1K), as reported previously [28, 30]. qPCR and western blot analysis showed that SFRP2 was highly expressed in cells from DM than cells from UM (Figure 1L,M). These results suggest that SFRP2 was positively correlated to metastasis of colon cancer and related to poor outcomes of patients with colon cancer.

### 3.2 SFRP2 Promotes Colon Cancer Cell Migration and Invasion but Has no Effect on Proliferation In Vitro

**FIGURE 2:**
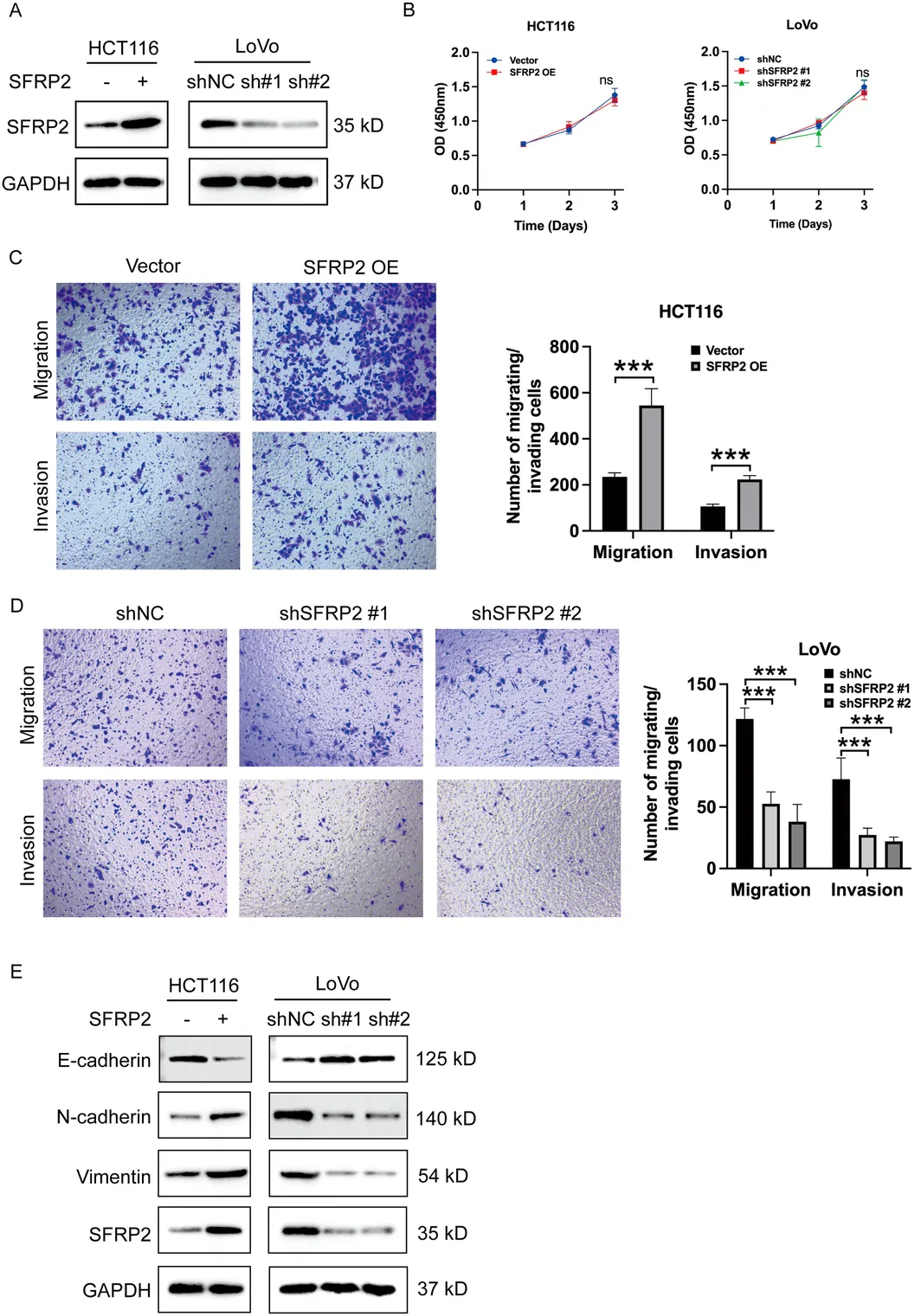
SFRP2 promotes migration and invasion but not proliferation in colon cancer cells. (A) Western blot analysis of SFRP2 in HCT116 cells transfected with SFRP2‐overexpression plasmid and LoVo cells transfected with shSFRP2s. (B) Effects of SFRP2 on proliferation of HCT116 and LoVo cells by CCK‐8 assays. (C) Effects of SFRP2 on migration of HCT116 and LoVo cells by Transwell assays. (D) Effects of SFRP2 on invasion of HCT116 and LoVo cells by Transwell‐Matrigel assays. (E) Epithelial‐mesenchymal transition related proteins such as E‐cadherin, N‐cadherin, Vimentin and SFRP2 were assessed in SFRP2‐overexpression and knockdown cell lines. ***p < 0.001.

Next, we investigated the role of SFRP2 in colon cancer in vitro. We performed gain‐of‐function experiments using SFRP2‐overexpressing HCT116 cells and loss‐of‐function experiments using SFRP2‐knockdown LoVo cells (Figure 2A). Cell counting kit‐8 assay showed that abnormal expression of SFRP2 had no effect on proliferation of colon cancer (Figure 2B). Transwell and transwell‐matrigel assays showed that overexpression of SFRP2 promoted cell migration and cell invasion whereas knockdown of SFRP2 emerged suppressed effects (Figure 2C,D). In addition, SFRP2 overexpression increased mesenchymal‐related proteins such as N‐cadherin and Vimentin expressions and decreased epithelial‐related protein E‐cadherin, which is just in contrast to the effects of SFRP2 knockdown. These data reveal that SFRP2 positively regulates cell migration, invasion and epithelial‐mesenchymal transition (EMT) in colon cancer cells.

### 3.3 SFRP2 Interacts With Snai1 to Improve the Protein Stability of Snai1

**FIGURE 3:**
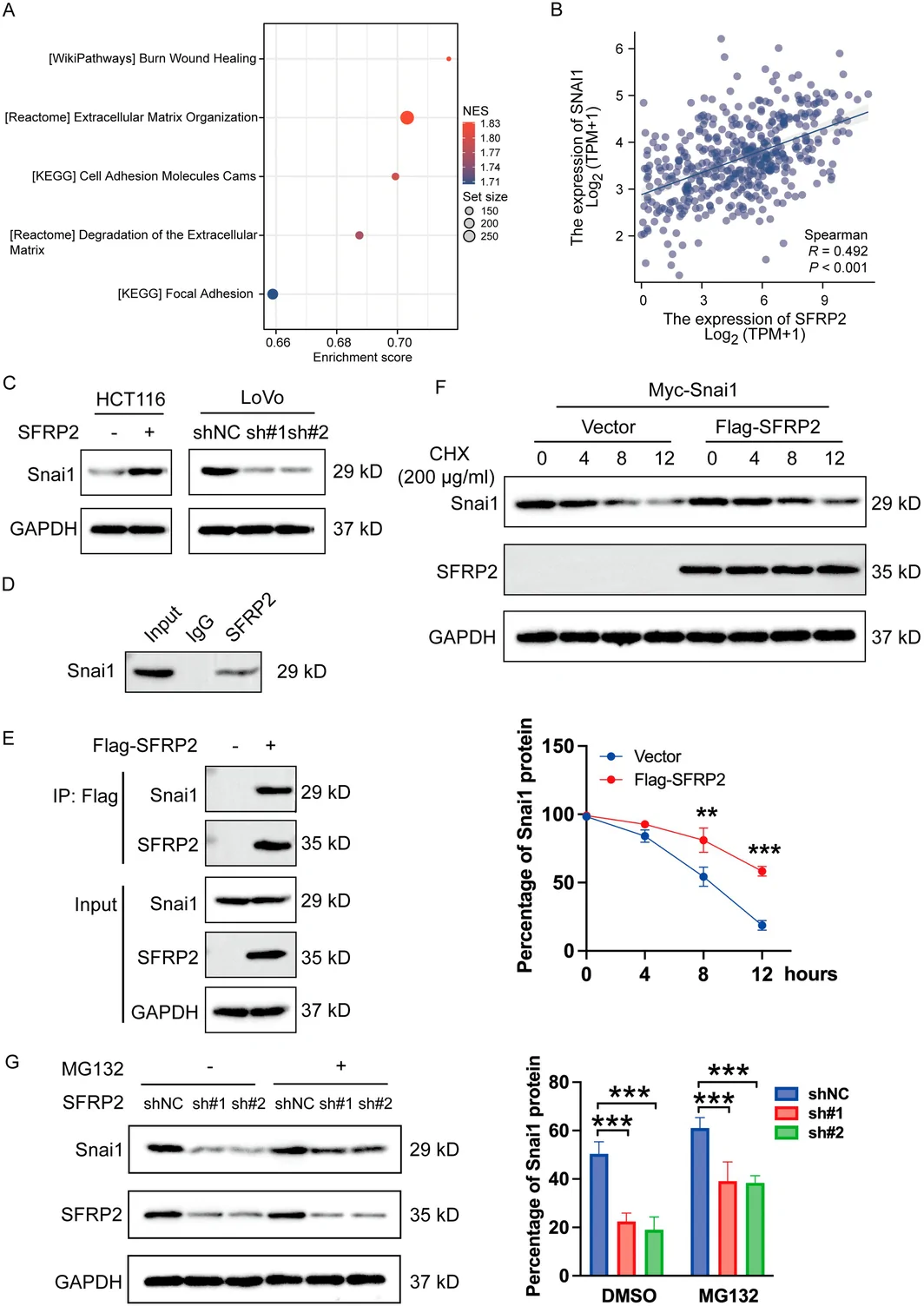
SFRP2 interacts with Snai1 protein to improve the stability of Snai1 protein. (A) GSEA analysis of SFRP2 co‐upregulated proteins. (B) Correlation analysis of SFRP2 and Snai1 in TCGA colon cancer database. (C) Western blot analysis of Snai1 expression in SFRP2‐overexpression and knockdown cell lines. (D) Endogenous immunoprecipitation of the Snai1 protein by an anti‐SFRP2 antibody in LoVo cells. (E) Immunoprecipitation of the Snai1 protein by an anti‐Flag antibody in HCT116 cells transfected with Flag‐SFRP2. Cells transfected with empty vector were used as a negative control. (F) HCT116 cells were transfected with Flag‐tagged SFRP2 expression plasmids and Myc‐tagged Snai1 expression plasmids then treated with CHX at the concentration of 200 μg/mL at 4, 8, 12 h. The protein degradation of Snai1 was assessed by western blot. (G) Western blot analysis of Snai1 expression in LoVo cells transfected with shSFRP2s with the treatment of MG132 at a concentration of 20 μM for 4 h. **p < 0.01, ***p < 0.001.

To explore the mechanism underlying the SFRP2 in colon cancer, we performed GSEA analysis of SFRP2. The enrichment pathway was mainly focused on focal adhesion, degradation of the extracellular matrix and cell adhesion molecules (Figure 3A) and Snai1 was a key protein in cell adhesion and EMT pathway. Pearson correlation analysis revealed that there was a positive relationship between SFRP2 and Snai1 (Figure 3B). Overexpression of SFRP2 increased Snai1 protein level while knockdown of SFRP2 reduced Snai1 expression (Figure 3C). To further confirm the interaction between SFRP2 and Snai1, we performed endogenous immunoprecipitation (IP) assay using Snai1‐specific antibody (Figure 3D). We found that Snai1 could strongly bind to SFRP2 (Figure 1D). What's more, we also did co‐IP in HCT116 cells by transfecting with Flag‐tagged SFRP2. The result indicated that there was an interaction between SFRP2 and Snai1 (Figure 1E). These findings suggest that SFRP2 binding to Snai1 regulated metastatic cell behaviours of colon cancer.

Next, we used a protein synthesis inhibitor Cycloheximide (CHX) to investigate whether SFRP2 affected Snai1 protein stability. Treatment of 200 μg/mL CHX for different times was applied to block protein synthesis. Western blotting assay showed that overexpression of SFRP2 significantly slowed down the Snai1 protein degradation compared to vector control (Figure 3F). Furthermore, we confirmed SFRP2‐mediated Snai1 protein degradation regulatory system using the 26S proteasome inhibitor MG132. It was observed that the Snai1 protein levels with MG132 treatment could not be further reduced in SFRP2 knockdown cells compared to non‐proteasome inhibitor treatment. Our data demonstrate that SFRP2 enhances Snai1 expression by maintaining its protein stability via the proteasome way.

### 3.4 SFRP2 Suppresses Snai1 Ubiquitination via Deubiquitinase USP11

**FIGURE 4:**
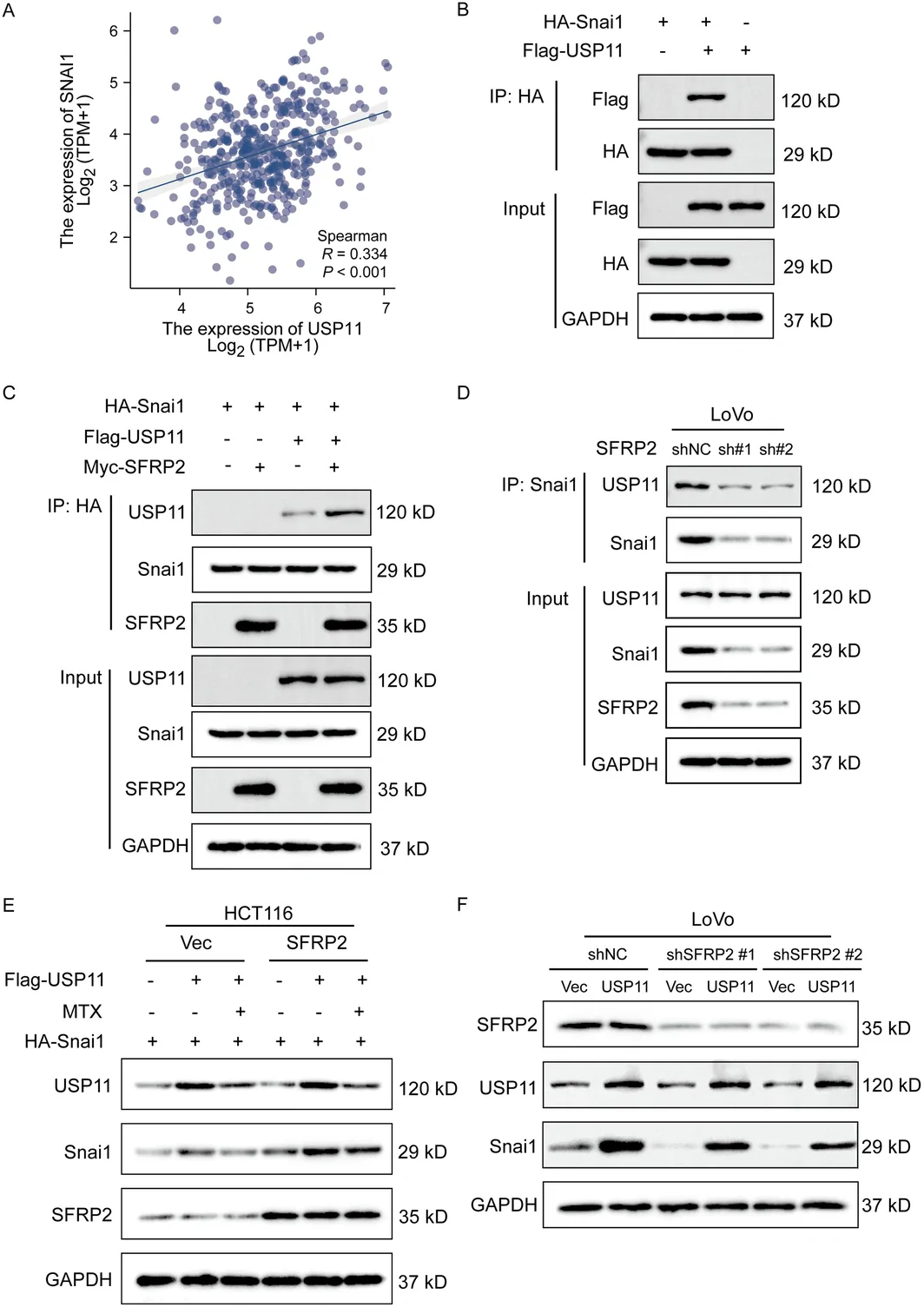
SFRP2 relieves Snai1 ubiquitination through deubiquitinase USP11. (A) Correlation analysis of SFRP2 and Snai1 in TCGA colon cancer database. (B) Immunoprecipitation of USP11 protein using anti‐HA antibody in HCT116 cells transfected with HA‐tagged Snai1 and Flag‐tagged USP11 plasmids. (C) Western blot analysis of SFRP2, Snai1 and USP11 after co‐immunoprecipitation in cells co‐transfected with HA‐tagged Snai1, Myc‐tagged SFRP2 and Flag‐tagged USP11. (D) Immunoprecipitation of USP11 using anti‐Snai1 antibody in LoVo cells transfected with shSFRP2s. (E) HCT116‐Vector/HCT116‐SFRP2 cells were transfected with vector, Flag‐USP11, Flag‐USP11 with treatment of USP11 inhibitor mitoxantrone (5 nmol/L). Western blot analysis of SFRP2, Snai1 and USP11 was measured in the above groups. (F) Western blot analysis of Snai1 expression in SFRP2‐knockdown LoVo cells transfected with vector or Flag‐USP11. GAPDH was used as a loading control.

To further investigate how SFRP2 affected Snai1 protein stability, we predicted that the deubiquitinase USP11 may potentially interact with Snai1 via Biogrid (https://thebiogrid.org/). Pearson correlation analysis showed that there was a positive relationship between USP11 and Snai1 (Figure 4A). The co‐IP result identified that USP11 could bind to Snai1 (Figure 4B). What's more, we transiently transfected Flag‐tagged USP11 and Myc‐tagged SFRP2 and performed IP using HA‐tagged Snai1. Results showed that in the absence of SFRP2, there was an interaction between Snai1 and USP11, of which the interaction became strong in the presence of SFRP2 (Figure 4C), indicating that SFRP2 promoted the binding of Snai1 to USP11. In addition, we further performed endogenous IP in the SFRP2‐knockdown cells. It was found that USP11 reduced the binding to Snai1 with the silence of SFRP2 (Figure 4D).

Ubiquitin‐specific peptidases (USPs), particularly USP11, play a critical role in various cellular processes by regulating protein stability through deubiquitination. The balance of ubiquitination and deubiquitination is pivotal in cellular homeostasis, impacting mechanisms such as apoptosis, DNA damage response, cell cycle progression and tumorigenesis [31, 32, 33, 34, 35]. Thus, we used USP11 inhibitor mitoxantrone (MTX) to inhibit the deubiquitinase activity of USP11 [36, 37] to verify that SFRP2 stabilizes Snai1 protein through the USP11 deubiquitinase activity. We found that the protein stability of Snai1 was better in the USP11‐expressing group than vector control group, while its protein stability was obviously attenuated in USP11‐expressing and MTX‐treated group. The effects were more reinforced with SFRP2 overexpression (Figure 4E). To further determine whether USP11 regulates Snai1 protein stability independently of SFRP2, we overexpressed USP11 in SFRP2‐knockdown LoVo cells. Western blot analysis revealed that USP11 overexpression significantly restored Snai1 protein levels despite the absence of SFRP2 (Figure 4F). These results indicate that USP11 is sufficient to stabilize Snai1 independently, while SFRP2 enhances this process by promoting the interaction between USP11 and Snai1. Thus, SFRP2 functions as a facilitator rather than an absolute requirement for USP11‐mediated Snai1 deubiquitination.

### 3.5 Snai1 Positively Regulates SFRP2 Transcription

**FIGURE 5:**
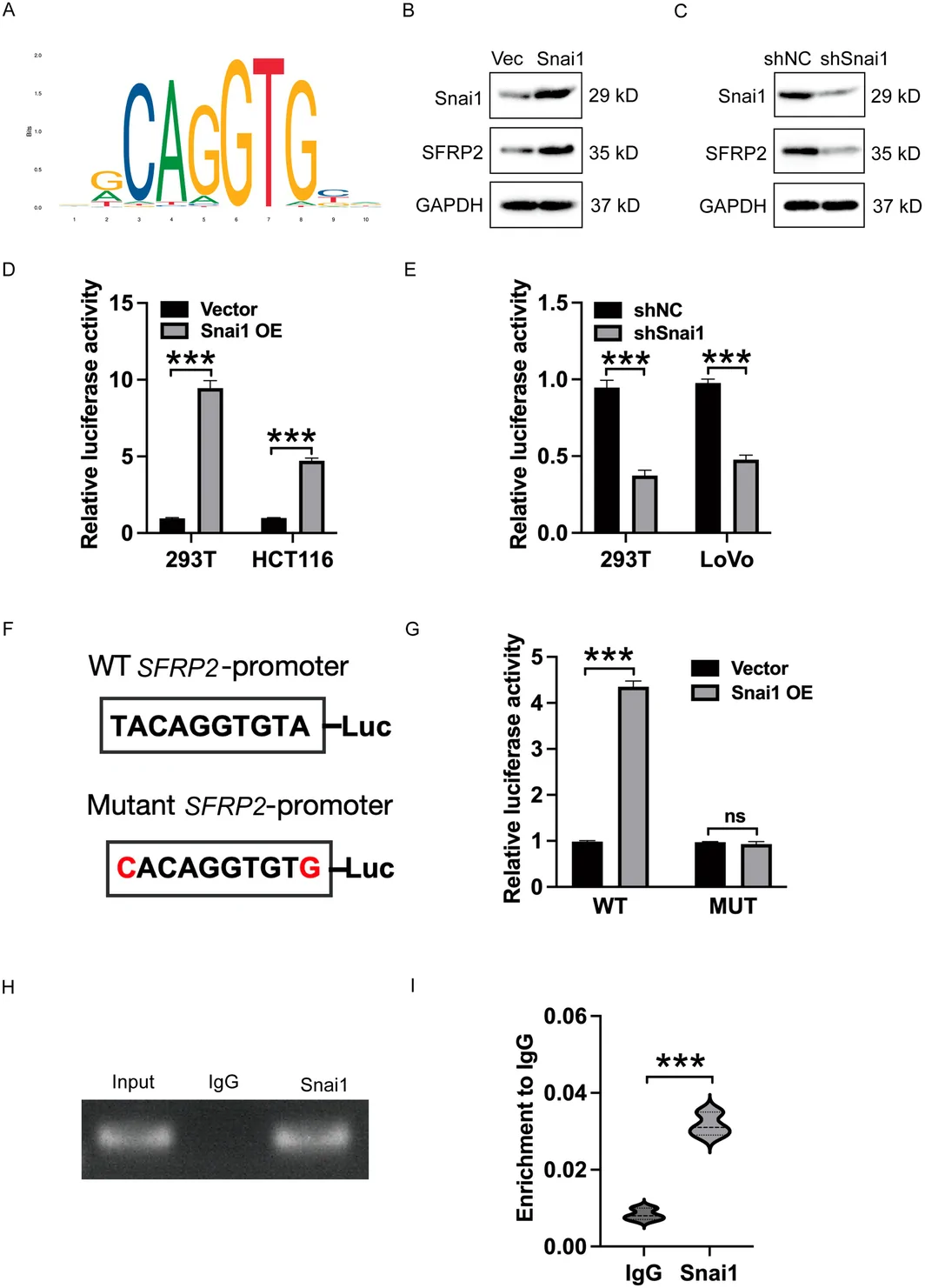
Snai1 directly bound to the SFRP2 promoter and increased SFRP2 transcription. (A) The predicted binding site of Snail1 in the promoter of SFRP2. (B‐C) Western blot analysis of SFRP2 in Snai1‐expression HCT116 cells and Snai1‐knockdown LoVo cells. (D) Luciferase reporter analysis of SFRP2‐promoter in Snai1‐expression HCT116 cells. (E) Luciferase reporter analysis of SFRP2‐promoter in Snai1‐knockdown LoVo cells. (F) The schematic of luciferase reporter plasmids of SFRP2 wildtype promoter and SFRP2 mutant promoter. (G) Luciferase reporter analysis of SFRP2‐promoter and SFRP2‐mutant‐promoter in HCT116 cells. (H) Agarose electrophoresis for ChIP analysis of Snai1 binding to the SFRP2 promoter. (I) qRT–PCR for ChIP analysis of Snai1 binding to the SFRP2 promoter. ***p < 0.001.

Subsequently, we found that there was a binding motif of Snai1 on SFRP2 promoter (Figure 5A) by JASPAR (https://jaspar.elixir.no/). We hypothesized that Snail1 may regulate SFRP2 expression at the transcription level. SFRP2 was up‐regulated in Snai1‐overexpressing cells while down‐regulated in Snai1‐knockdown cells (Figure 5B,C). Consistently, the luciferase reporter assays showed that the reporter activity of SFRP2 promoter was elevated by over‐expression of Snail1 while suppressed by knockdown of Snai1 (Figure 5D,E). Next, we constructed the specific mutant luciferase reporter plasmid (Figure 5F). However, Snail1 could not increase the STIM1 promoter containing mutant binding sites activity (Figure 5G). Furthermore, ChIP assays validated that Snai1 could directly bind to the SFRP2 promoter (Figure 5H,I). The above results show that Snai1 regulates SFRP2 expression by directly binding the SFRP2 promoter. Taken together, these data indicate that there is a positive feedback regulatory network between SFRP2 and Snai1 in colon cancer.

### 3.6 SFRP2‐Snai1 Promotes Colon Cancer Migration and Invasion via PI3K/AKT/mTOR Pathway

**FIGURE 6:**
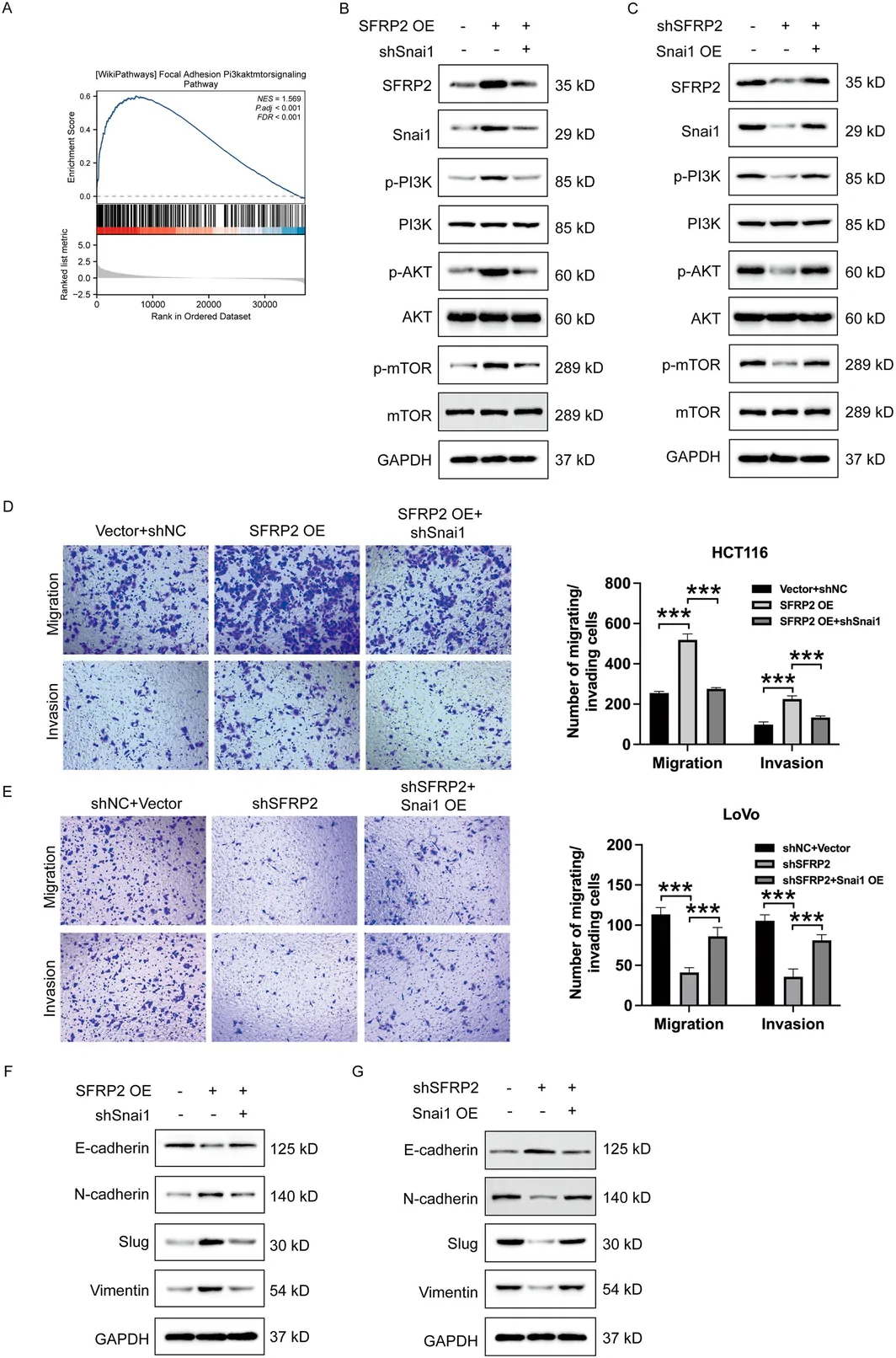
The SFRP2‐Snai1 axis promotes PI3K/AKT/mTOR pathway in colon cancer cells. (A) Gene set enrichment analysis of SFRP2 in PI3K/Akt signalling pathway. (B) Western blot analysis of PI3K, p‐PI3K, Akt, p‐Akt, mTOR, p‐mTOR in HCT116‐vector and HCT116‐SFRP2 treated with shSnai1 or shNC or LoVo‐shNC and LoVo‐shSFRP2 treated with transient transfection of Snai1 overexpression plasmids. (C and D) Effects of SFRP2‐Snai1 on migration of HCT116 and LoVo cells by Transwell assays. (E and F) Effects of SFRP2‐Snai1 on invasion of HCT116 and LoVo cells by Transwell‐matrigel assays. (G and H) Effects of SFRP2‐Snai1 on EMT‐related proteins such as E‐cadherin, N‐cadherin, Vimentin were assessed by western blot. ***p < 0.001.

We wondered through which signalling pathway SFRP2‐Snai1 axis in promoting colon cancer cell migration and invasion, GSEA analysis showed that the SFRP2 related gene sets enriched in PI3K/AKT/mTOR signalling pathway (Figure 6A). We used HCT116‐SFRP2 cells with or without shSnai1 and LoVo‐shSFRP2 cells with or without Snai1‐ovexpression to evaluate the expression of the PI3K/AKT/mTOR pathway. SFRP2 overexpression or knockdown could promote or suppress the phosphorylation levels of PI3K, AKT and mTOR, while knockdown or overexpression of Snai1 could reverse the corresponding effects (Figure 6B). To further clarify whether PI3K signalling is activated downstream of the SFRP2–Snai1 axis, we treated SFRP2‐overexpressing HCT116 cells with the PI3K inhibitor LY294002. Western blot analysis showed that LY294002 treatment effectively suppressed SFRP2‐induced PI3K phosphorylation, whereas total PI3K levels remained unchanged (Figure S1). Importantly, neither Snai1 nor SFRP2 protein expression was affected by PI3K inhibition. These results suggest that activation of PI3K signalling occurs downstream of the SFRP2–Snai1 axis and does not regulate Snai1 expression, supporting a unidirectional signalling cascade from SFRP2–Snai1 to PI3K activation. What's more, we further verified the role of SFRP2‐Snai1 in colon cancer migration, invasion and EMT. In SFRP2‐overexpressing cells, the migratory and invasive abilities of HCT116 cells were increased, after Snai1 was knockdown, the enhanced effects were partially blocked (Figure 6C–F). In SFRP2‐knockdown cells, the migratory and invasive abilities of LoVo cells were attenuated, after Snai1 was upregulated, the suppressed effects were rescued (Figure 6C–F). Consistent with the above results, SFRP2 overexpression‐induced the upregulation of the mesenchymal‐related proteins N‐cadherin, Slug and Vimentin could be reversed by knockdown of Snai1 (Figure 6G). Oppositely, SFRP2 knockdown‐induced the downregulation of N‐cadherin, Slug and Vimentin could be rescued by overexpression of Snai1 (Figure 6H). These results reveal that SFRP2 promotes colon cancer cell migration, invasion and EMT through Snai1 via the PI3K/Akt/mTOR pathway in vitro.

### 3.7 SFRP2‐Snai1 Promotes Metastasis In Vivo

**FIGURE 7:**
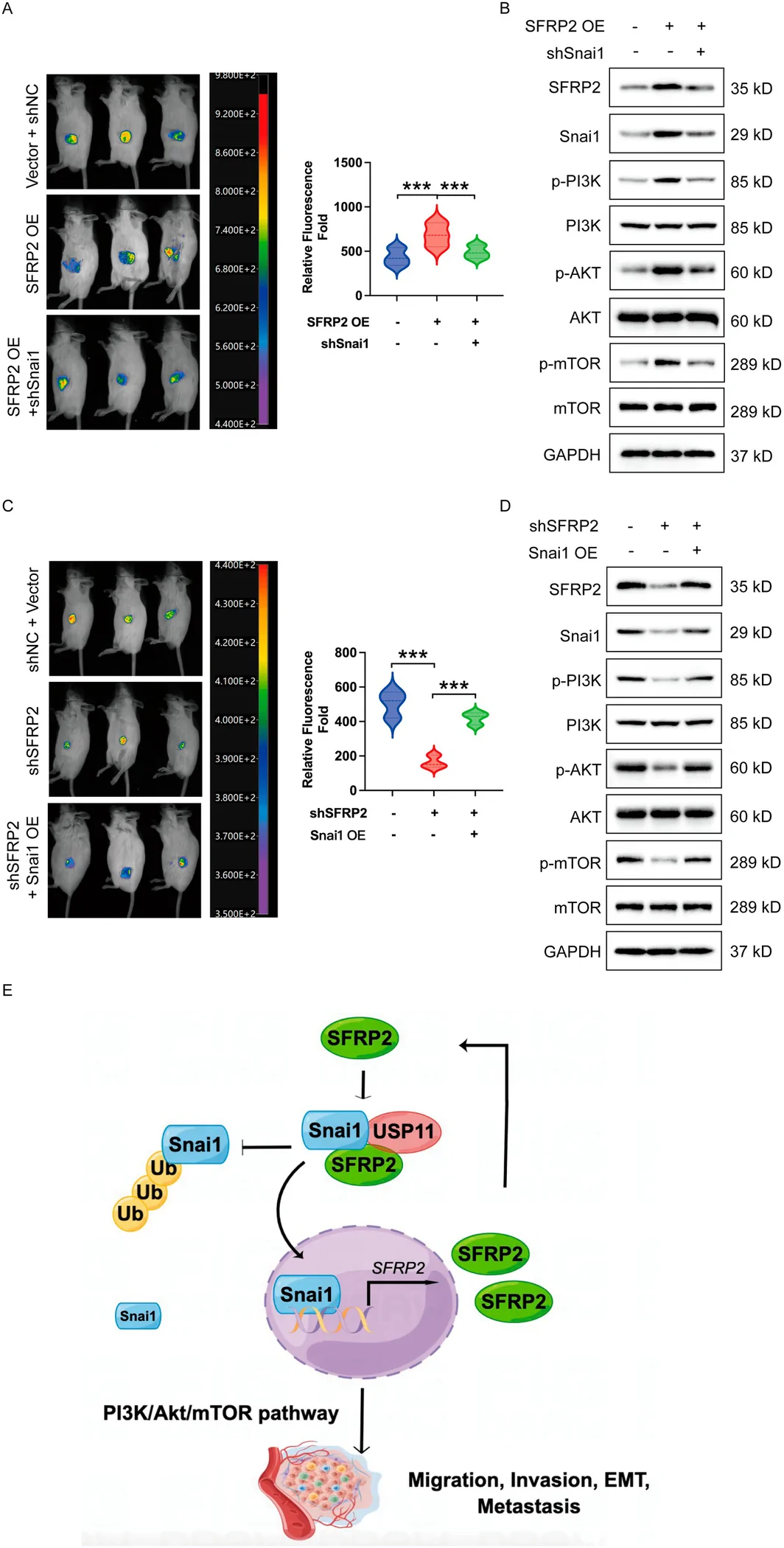
SFRP2 promotes metastasis in vivo. (A) Representative bioluminescence images of NSG mice injected with SFRP2‐expression with or without Snai1‐knockdown stable HCT116 cells. (B) Western blot analysis of expression of PI3K, p‐PI3K, Akt, p‐Akt, mTOR, p‐mTOR, Snai1, SFRP2 of 3 groups of tumour‐bearing mice. (C) Representative bioluminescence images of NSG mice injected with SFRP2‐knockdown with or without Snai1 expression stable LoVo cells. (D) Western blot analysis of expression of PI3K, p‐PI3K, Akt, p‐Akt, mTOR, p‐mTOR, Snai1, SFRP2 of the above 3 groups of tumour‐bearing mice. (E) The schematic illustration of SFRP2/Snai1/USP11 positive feedback loop promoting colon cancer metastasis via the PI3K/Akt/mTOR pathway. ***p < 0.001.

Lastly, we evaluated the biological function of SFRP2‐Snai1 in colon cancer metastasis in vivo. SFRP2‐overexpressing cells (HCT116‐SFRP2), the corresponding controls (HCT116‐Control) and HCT116‐shSnai1 cells, SFRP2‐knockdown cells (LoVo‐shSFRP2), the corresponding controls (LoVo‐Control) and LoVo‐Snai1 cells were injected into the lateral tail veins of nude mice, respectively. The number of liver tumour metastasis was remarkably increased in the SFRP2 overexpression group compared to the control group, and the additive treatment with shSnai1 reversed the role of SFRP2 (Figure 7A). The ex vivo immunoblot analysis showed that SFRP2 overexpression enhanced the expressions of SFRP2, Snai1 and phosphorylated PI3K, AKT and mTOR in xenograft tumour tissues, and Snai1 knockdown could block the effects (Figure 7B). Contrary, mice injected with SFRP2‐knockdown cells showed less liver metastasis and decreased phosphorylation levels of PI3K/AKT/mTOR pathway, which was rescued by Snai1 overexpression (Figure 7C,D).

To sum up, these results demonstrate that SFRP2 can promote colon cancer metastasis in vivo through Snai1 via PI3K/AKT/mTOR pathway.

## 4 Discussion

SFRP2 has been implicated in enhancing cellular migration and invasion, rather than proliferation, indicating its specific contribution to metastatic behaviour. Mechanistically, SFRP2 interacts with Snai1, a key transcription factor associated with epithelial‐mesenchymal transition (EMT), to stabilize Snai1 protein levels. This interaction is further facilitated by the deubiquitinase USP11, which alleviates Snai1 ubiquitination, thereby promoting its stability and activity. Notably, Snai1 has been shown to bind directly to the SFRP2 promoter, creating a feedback loop that enhances SFRP2 transcription. This regulatory axis not only contributes to the transcriptional activation of SFRP2 by Snai1 but also promotes the activation of the PI3K/AKT/mTOR pathway, which is crucial for cancer cell survival and metastasis. Collectively, these findings elucidate the pivotal role of SFRP2 in colon cancer progression and highlight its potential as a therapeutic target in metastatic colon cancer.

SFRP2 is a member of the SFRP family, which plays a crucial role in modulating Wnt signalling pathways [38]. SFRP2 has been implicated in various biological functions, including cell adhesion, migration and invasion, particularly in certain cancer [39, 40, 41, 42, 43]. In this study, SFRP2 is notably overexpressed in metastatic colon cancer tissues and TGF‐β‐induced cancer cells and correlates with poor prognosis of colon cancer patients, highlighting its potential as a biomarker for aggressive disease. Furthermore, this study also indicates that SFRP2 enhances the migratory and invasive capabilities of colon cancer cells without significantly affecting cell proliferation in vitro.

Mechanistically, SFRP2 interacts with Snai1, a transcription factor known for its role in epithelial‐mesenchymal transition (EMT) [44, 45], thereby stabilizing Snai1 protein levels. This interaction is further facilitated by SFRP2's ability to suppress Snai1 ubiquitination through the deubiquitinase USP11, which prevents Snai1 degradation. Interestingly, Snai1 also positively regulates the transcription of SFRP2, creating a feedback loop that amplifies their cooperative effects on cancer cell behaviour. The SFRP2‐Snai1 axis has been shown to promote colon cancer migration and invasion via the phosphoinositide 3‐kinase (PI3K)/AKT/mTOR signalling pathway, underscoring its significance in tumour progression. In vivo studies further confirm that the SFRP2‐Snai1 interaction contributes to metastasis, suggesting that targeting this pathway may offer therapeutic potential in managing metastatic colon cancer. Despite these insights, further research is needed to fully elucidate the complexities of SFRP2's role in cancer biology and its potential as a therapeutic target.

Ubiquitin‐specific protease 11 (USP11) is a deubiquitinating enzyme that plays a significant role in various cellular processes, including signal transduction, gene regulation, cell proliferation and apoptosis [33, 46, 47, 48, 49]. Given its involvement in malignancies and cellular stress responses, USP11 has garnered attention as a potential biomarker and therapeutic target across multiple cancers, including prostate, colorectal and hepatic cancers [50, 51, 52, 53, 54]. Recent studies highlight its dual role as both a promoter of oncogenic signalling pathways and a modulator of cellular responses to stressors, including chemotherapy and radiation. USP11‐mediated LSH deubiquitination inhibits ferroptosis in colorectal cancer via epigenetic activation of CYP24A1 [32] USP11 potentiates HGF/AKT signalling and drives metastasis in hepatocellular carcinoma [33] USP11 regulates autophagy‐dependent ferroptosis after spinal cord ischemia–reperfusion injury by deubiquitinating Beclin 1 [35] USP11 enhances TGFβ‐induced epithelial‐mesenchymal plasticity and human breast cancer metastasis [45]. This work identified that SFRP2 could recruit USP11 to stabilize Snai1 protein, thus preventing Snai1 ubiquitination and facilitate colon cancer metastasis.

Importantly, the SFRP2‐Snai1 complex activates the PI3K/AKT/mTOR signalling pathway, which is well‐established in mediating cellular processes associated with cancer metastasis. The activation of this pathway underscores the functional significance of the SFRP2‐Snai1 interaction in enhancing metastatic potential, as demonstrated in vivo. Collectively, these findings elucidate a critical molecular‐pathway relationship that not only contributes to our understanding of colon cancer metastasis but also presents potential therapeutic targets for intervention.

The limitations of this research warrant consideration, particularly regarding the focus on the SFRP2‐Snai1 axis, which may not wholly represent the complexity of metastatic colon cancer. The reliance on in vitro models raises questions about the translational applicability of the findings, as tumour microenvironments and intercellular interactions are not fully replicated. Additionally, while the study elucidates mechanisms of migration and invasion, the potential roles of other signalling pathways or factors in metastasis remain to be explored, necessitating a broader approach in future research.

In conclusion, the findings demonstrate that SFRP2 is elevated in metastatic colon cancer and correlates with unfavourable prognosis, highlighting its role as a critical factor in colon cancer progression and metastasis. SFRP2 enhances colon cancer cell migration and invasion while not affecting proliferation in vitro. Mechanistically, SFRP2 stabilizes Snai1 by preventing its ubiquitination through the deubiquitinase USP11, subsequently facilitating positive feedback that elevates SFRP2 transcription. The SFRP2‐Snai1 axis activates the PI3K/AKT/mTOR pathway, promoting metastatic behaviour in vivo (Figure 7E). This research underscores the significance of the SFRP2‐Snai1 interaction as a pivotal mechanism underlying colon cancer metastasis.

## Author Contributions

**Yanshen Kuang:** data curation (equal), formal analysis (equal), investigation (equal), writing – original draft (equal). **Xin Liu:** conceptualization (equal), data curation (equal), formal analysis (equal), investigation (equal). **Mu Ke:** data curation (supporting), formal analysis (supporting), methodology (lead). **Zhijie Chang:** formal analysis (supporting), methodology (supporting), validation (equal). **Baoqing Jia:** funding acquisition (lead), methodology (supporting), validation (supporting). **Bing Li:** project administration (supporting), supervision (equal), writing – review and editing (equal). **Honggang Qian:** project administration (lead), supervision (equal), writing – review and editing (equal).

## Funding

This work was supported by the National Nature Science Foundation of China (No. 82173355).

## Conflicts of Interest

The authors declare no conflicts of interest.

## Supporting information


**Figure S1:** PI3K inhibition suppresses SFRP2‐induced PI3K phosphorylation without affecting Snai1 expression. Western blot analysis of PI3K, phosphorylated PI3K (p‐PI3K), Snai1 and SFRP2 in SFRP2‐overexpressing HCT116 cells treated with LY294002. GAPDH was used as a loading control.

**Table S1:** Sequences of qPCR primers.

## Data Availability

Data Availability Data are available from the corresponding author upon reasonable request.
