# Supplementary material for: SFRP2 Potentiates Metastasis of Colon Cancer by Enhancing Snai1 Protein Stability via USP11
Source: J Cell Mol Med. 2026 Sep 11;30(17):e71318. doi: 10.1111/jcmm.71318 (PMC13564241; doi:10.1111/jcmm.71318)
Supplement: Supplementary file 1 [file JCMM-30-e71318-s001.docx]

Supplementary Figures

Supplementary Figure S1. PI3K inhibition suppresses SFRP2-induced PI3K phosphorylation without affecting Snai1 expression. Western blot analysis of PI3K, phosphorylated PI3K (p-PI3K), Snai1, and SFRP2 in SFRP2-overexpressing HCT116 cells treated with LY294002. GAPDH was used as a loading control.
