# Supplementary material for: SFRP2 Potentiates Metastasis of Colon Cancer by Enhancing Snai1 Protein Stability via USP11
Source: J Cell Mol Med. 2026 Sep 11;30(17):e71318. doi: 10.1111/jcmm.71318 (PMC13564241; doi:10.1111/jcmm.71318)
Supplement: Supplementary file 2 [file JCMM-30-e71318-s002.docx]

Supplementary Table 1 Sequences of qPCR primers

| Gene | Forward (5’-3’) | Reverse (5’-3’) |
| --- | --- | --- |
| SFRP2 | ACGGCATCGAATACCAGAACA | CTCGTCTAGGTCATCGAGGCA |
| GAPDH | GGAGCGAGATCCCTCCAAAAT | GGCTGTTGTCATACTTCTCATGG |

| shRNAs | | Sequences |
| --- | --- | --- |
| NC | Oligo 1 | 5'-GATCCTTCTCCGAACGTGTCACGTCTCGAGACGTGACACGTTCGGAGAATTTTTTG-3' |
|  | Oligo 2 | 5'-AATTCAAAAATTCTCCGAACGTGTCACGTCTCGAGACGTGACACGTTCGGAGAAG-3' |
| SFRP2 #1 | Oligo 1 | 5'-GATCCATGATGATGACAACGACATAACTCGAGTTATGTCGTTGTCATCATCATTTTTTTG-3' |
|  | Oligo 2 | 5'-AATTCAAAAAATGATGATGACAACGACATAACTCGAGTTATGTCGTTGTCATCATCATG-3' |
| SFRP2 #2 | Oligo 1 | 5'-GATCCGACCAAGAGCAAGACCATTTACTCGAGTAAATGGTCTTGCTCTTGGTCTTTTTTG-3' |
|  | Oligo 2 | 5'-AATTCAAAAAGACCAAGAGCAAGACCATTTACTCGAGTAAATGGTCTTGCTCTTGGTCG-3' |
| Snai1 #1 | Oligo 1 | GATCCAGCGAGCTGCAGGACTCTAATCTCGAGATTAGAGTCCTGCAGCTCGCTTTTTTG |
|  | Oligo 2 | AATTCAAAAAAGCGAGCTGCAGGACTCTAATCTCGAGATTAGAGTCCTGCAGCTCGCG |
| Snai1 #2 | Oligo 1 | GATCCGAAAGGCCTTCAACTGCAAATCTCGAGATTTGCAGTTGAAGGCCTTTCTTTTTTG |
|  | Oligo 2 | AATTCAAAAAGAAAGGCCTTCAACTGCAAATCTCGAGATTTGCAGTTGAAGGCCTTTCG |
